# A Role of Intracellular Toll-Like Receptors (3, 7, and 9) in Response to *Mycobacterium tuberculosis* and Co-Infection with HIV

**DOI:** 10.3390/ijms21176148

**Published:** 2020-08-26

**Authors:** Huy Nguyen, Nicky Gazy, Vishwanath Venketaraman

**Affiliations:** 1 College of Osteopathic Medicine of the Pacific, Western University of Health Sciences, Pomona, CA 91766-1854, USA; huy.nguyen@westernu.edu; 2 Beaumont Health System, 5450 Fort St, Trenton, MI 48183, USA; nicky.gazy@gmail.com

**Keywords:** Toll-like receptor 3, Toll-like receptor 7, Toll-like receptor 9, HIV, *Mycobacterium tuberculosis*

## Abstract

*Mycobacterium tuberculosis* (Mtb) is a highly infectious acid-fast bacillus and is known to cause tuberculosis (TB) in humans. It is a leading cause of death from a sole infectious agent, with an estimated 1.5 million deaths yearly worldwide, and up to one third of the world’s population has been infected with TB. The virulence and susceptibility of Mtb are further amplified in the presence of Human Immunodeficiency Virus (HIV). Coinfection with Mtb and HIV forms a lethal combination. Previous studies had demonstrated the synergistic effects of Mtb and HIV, with one disease accelerating the disease progression of the other through multiple mechanisms, including the modulation of the immune response to these two pathogens. The response of the endosomal pattern recognition receptors to these two pathogens, specifically toll-like receptors (TLR)-3, -7, and -9, has not been elucidated, with some studies producing mixed results. This article seeks to review the roles of TLR-3, -7, and -9 in response to Mtb infection, as well as Mtb-HIV-coinfection via Toll-interleukin 1 receptor (TIR) domain-containing adaptor inducing INF-β (TRIF)-dependent and myeloid differentiation factor 88 (MyD88)-dependent pathways.

## 1. TB Pathogenesis and Host Immune Response

Tuberculosis (TB) is a highly contagious and airborne infectious disease caused by *Mycobacterium tuberculosis* (Mtb), an acid-fast bacillus. Mtb has been coexisting and coevolving with humans throughout history since ancient times [1]. The interaction of Mtb and host immune defense can result in three possible outcomes: (1) the control of infection, (2) the development of active disease, or (3) latent infection [2]. In 2018, TB was the leading cause of death worldwide from a sole infectious agent, with up to one-third of the world’s population being infected with TB [3]. However, the number of latent TB is not precisely known. Patients with latent TB have an overall of 5–10% lifetime risk of succumbing to the reactivation of TB [3]. Owing to their transmission route, Mtb will first encounter innate immune cells, particularly macrophages and dendritic cells residing in the human lungs [4,5]. Upon encounter, the pattern recognition receptors (PRRs) present on the surface of these immune cells will recognize and bind to pathogen-associated molecular patterns (PAMPs) expressed on the Mtb cell wall [6]. Among the families of PRR, toll-like receptors (TLRs), C-type lectin receptors (CLRs), and nucleotide-binding oligomerization (NOD)-like receptors (NLRs), are known to play critical roles in recognizing and uptaking Mtb [7]. The formation of the ligand–receptor complex will result in the phagocytosis of Mtb in host immune cells and subsequently form phagosome–lysosome fusion. The fusion can facilitate Mtb clearance by mounting innate immune responses via the production of cytokines and chemokines, the induction of autophagy [8], and the presentation of bacterial antigen to T-helper cells for an adaptive immunity response [6]. The protective roles of TLR signaling against Mtb remain controversial. For instance, studies have found that mice lacking TLR2 and TLR9 had a markedly increased susceptibility and succumb more rapidly to Mtb infection comparing to wild-type mice [9,10,11]. Conversely, Christoph et al. reported no difference in pro-inflammatory cytokines or survival rates in TLR2/TLR4/TLR9-deficient mice. Besides TLRs, NLRs, particularly NOD2, have been implicated in the activation of inflammatory pathways, decreasing bacterial burden, and recruiting autophagy-associated proteins in response to Mtb infection [12]. Multiple CLRs—namely, mannose receptor (MR), Mincle, Dectin-1, Dectin-2, Dectin-3, and CL-LK receptors—have been associated with host defense mechanisms against Mtb [8]. For example, MR, Dectin-2, and CL-LK recognize bacterial mannose-capped lipoarabinomannan (manLAM) and initiate the phagocytosis of Mtb by human macrophages [13,14,15]. Furthermore, both Mincle and Dectin-3 are necessary to induce immune responses against Mtb trehalose 6 and 6′-dimycolate, also known as cord factor. Mincle expression is also positively regulated by Dectin-3, thereby further augmenting the Mincle-mediated pathway [16,17]. Dectin-1, on the other hand, is not known to improve host survivability against Mtb. In fact, Dectin-1 makes a minor contribution to Mtb susceptibility in mice [18]. Interestingly, the cooperation of Dectin-1 and TLR2, which represents the cooperation between two PRR families, mediates the pro-inflammatory responses in macrophages following Mtb infection [19]. Despite the sophistication and redundancy of the host immune system, Mtb has developed multiple strategies to circumvent host cell defenses. For example, protein tyrosine phosphatase (PtpA), a protein secreted by Mtb, interferes with the host vacuolar-H (+)-ATPase pump, thereby inhibiting the acidification process of the phagosome [20]. Moreover, many studies have found that Mtb interferes with phagosome–lysosome fusion by the binding of bacterial mannose-capped lipoarabinomannan (manLAM) to the host mannose receptor (MR) [13] and causing the rupture of Mtb-contained phagosome by a type VII secretion system named ESX-1, resulting in the release of Mtb into the cytosol [21]. Jamwal et al. have discovered that, in addition to ESX-1, Mtb also activates the host cytoplasmic phospholipase A2 (cPLA2), an essential enzyme in phagosomal trafficking and the export of cargo from multiple intracellular compartments [22], culminating in the inhibition of macrophage apoptosis and the promotion of Mtb phagosome-lysosomal escape [23]. Intriguingly, Schafer et al. have found that the phagocytotic process of Mtb during primary infection is mostly through non-opsonic mechanisms, which are associated with the impairment of phagosome–lysosome fusion due to the lack of adaptive response. Conversely, in the later stage, the phagocytic process is mostly through the opsonized mechanism; thus, they do not inhibit phagosome–lysosome fusion [13]. Finally, many pieces of evidence support the idea that Mtb could disrupt the host autophagy process, thus enabling its intracellular survival via the expression of microRNA (miRNA)-33 [24], miRNA-27a [25], and miRNA-144 [26].

## 2. HIV Pathogenesis and Host Immune Response

Tuberculosis is a leading cause of death in the human immunodeficiency virus (HIV)-infected population [27]. HIV-infected individuals have a higher chance, as high as 19%, of contracting primary TB, and have a risk of latent TB reactivation of up to 10% per year [27]. HIV is an enveloped RNA virus that belongs to the family of Retroviridae, subfamily Orthoretrovirinae, and genus Lentivirus [28]. There are two strains of HIV, which are HIV-1 and HIV-2. Despite sharing similar genomic, structural, and antigenic characteristics with HIV-1, HIV-2 has a significantly lower pathogenesis capability and lower virulence [29]. As a result, most studies were directed at the pathogenesis of and host immune response against HIV-1. However, as part of their pathogenesis, both strains can induce chronic immune system activation and the destruction of immune cells, ultimately leading to acquired immunodeficiency syndrome (AIDS) [30].

Once introduced into the host, HIV infects various cell types, particularly immune cells such as CD4+ T cells, macrophages, and dendritic cells. The infection of HIV-1 in macrophages and dendritic cells is relatively unfavorable due to low triphosphate levels and the presence of reverse transcriptase inhibitor, SAMHD1 [31,32]. However, a recent study has revealed that SAMHD1 also suppresses the innate immune responses to HIV-1 infection by disrupting the NF-kB and type I interferons (IFNs) pathways [33]. The infection process is initiated with the binding of viral glycoprotein to host receptor CD4 with the sequential docking of the chemokine coreceptor CCR5 or CXCR4 [30,34]. This interaction prompts the fusion of the virus–host membrane and allows the entry of viral capsid into the cytosol. Once inside the cytosol, the viral RNA genome will use its reverse transcriptase to convert its RNA into viral DNA and initiate the degradation of viral capsid to release viral DNA into the cytosol, which is subsequently translocated into the host nucleus [34]. However, which of the two processes is initiated first is not well documented. Finally, the viral DNA will be integrated into the host genome using its integrase enzyme and become latent [35].

In the acute phase of the infection, the presence of HIV will lead to the activation of the innate immune cells, particularly macrophages and dendritic cells, through the interaction of host PRRs and viral PAMPs, which results in the production of type I IFNs, a cytokine responsible for restricting viral replication and transmission [36]. The secreted type I IFNs bind their receptors on uninfected cells to propagate the antiviral response [33]. Among many HIV-recognized PRRs that have been identified and investigated, TLR7/TLR8 and retinoic acid-inducible gene I (RIG-I)/melanoma-differentiation-associated protein 5 (MDA5), which bind to HIV-1 ssRNA, stimulate a profound antiviral inflammatory response [37,38,39,40]. Moreover, the HIV-1 reverse transcription intermediates—namely, cDNA, ssDNA, DNA/RNA hybrids, and dsDNA—also contribute to host innate immune antiviral responses through the activations of various cytoplasmic DNA sensors with the generation of type I IFNs [41]. Interestingly, in the presence of viral reverse transcriptase inhibitors, the host innate immune response is altered; thus, it is speculated that viral reverse transcriptase is also responsible for the differential effects on the host immune response [35,42]. The binding of PRRs and PAMPs also results in IL-15 production, a cytokine responsible for activating natural killer (NK) cells [43]. NK cells are critical immune cells in recognizing and destroying infected cells via two mechanisms: killer immunoglobulin receptors and killer immunoglobulin receptor-like molecules [43,44,45]. Surprisingly, a growing body of evidence has revealed the presence of reverse transcription products could potentially lead to a reduction in type I IFN production in macrophages [46]; evade the viral recognition of dendritic cells; induce an immunosuppressive state in infected DCs [47]; or stimulate the production of cytokine IL-1β from infected cells with the induction of cell death, particularly CD4+ T cells [48]. During the later stage of infection, HIV primarily targets HIV-specific CD4+ T cells during the CD4+ T cell proliferation and transition phase from naïve to effector and memory T cells [49,50], becoming HIV latent reservoirs. Collectively, HIV infection leads to the disturbance of CD4+ T cell functioning [42] and depletion in the number of CD4+ T cells [48]. As a cascade effect, the suppression of CD4+ T cell functioning and number can lead to B cell dysfunction and diminish the antibody response [42]. Moreover, the invasion of HIV initially leads to the proliferation and activation of HIV-specific cytotoxic CD8+ T cells [42]. Of note, several studies have noticed an inverse relationship between HIV-specific CD8+ T cells and viremia and seeding reservoir cells [51], and the influence of CD8+ T cells on the virus set point [52]. However, as infection and pro-inflammatory immune activation persist, HIV-specific CD8+ T cells become exhausted, with the hallmark loss of their effector functions and weakened capacity to destroy infected cells [53]. Most notably, a high level of programmed cell death protein 1 (PD-1) is expressed on HIV-specific T cells in HIV infection, and this is correlated with the impairment of the function of both CD8+ and CD4+ T cells [54,55]. Thus, PD-1 was hypothesized to be a primary marker of exhaustion and disease advancement [53,56]. Additionally, regulatory T (Treg) cells play a role in the pathogenesis and disease progression of HIV infection. Veiga-Parga et al. had found a high concentration of Tregs at sites of chronic HIV infection with an elevated production of IL-10, an anti-inflammatory cytokine with T-cell suppressive function [57]. As a result, the detrimental effects of persistent activation in response to chronic HIV infection by the host immune system create a window of opportunity for many pathogen invasions or reactivations, especially Mtb.

## 3. HIV-Mtb Coinfection and Host Immune Response

Studies have shown that HIV and Mtb coinfection have synergistic and cooperative effects, as one disease accelerates the progression of the other through various mechanisms. Mehto et al. found a significant up-regulation of anti-apoptotic proteins in Mtb-HIV-coinfection macrophages via the Toll-like receptor 2 (TLR-2) pathway, thereby promoting both pathogens’ survival [58]. Additionally, Mtb-HIV coinfection leads to the inhibition and downregulation of the antigen-presenting process within DCs. For example, Mtb inhibits Major Histocompatibility Complex Class II-HIV antigen processing and presentation in dendritic cells, hence promoting HIV-1 infection [59]. Similarly, coinfection with HIV downregulates Mtb-induced pro-inflammatory cytokine production and Mtb-induced MHC class II in DCs, which was not observed in HIV infection alone [60]. Several pieces of evidence establish the theory that HIV exacerbates Mtb pathogenesis, including the disruption of innate and adaptive immune functioning inside Mtb granuloma due to the recruitment of HIV+ cells to the periphery of the granuloma [61]; the impairment of the intracellular killing of Mtb in alveolar macrophages in Mtb and HIV-coinfected patients due to the interruption of the acidification of the Mtb-containing phagosome [62]; the impairment of CD4+ and CD8+ T cell responses to Mtb both at the periphery and within granulomas [63]; and the depletion of Mtb-specific CD4+ T cells before the depletion of generalized CD4+ T cells in HIV-infected individuals [64,65]. Furthermore, as mentioned above, chronic HIV infection induces T cell exhaustion and eventually ruins their ability to produce IFN-γ, a potent cytokine in effectively controlling Mtb infection [66,67]. There is also growing evidence that Mtb promotes HIV replication and infectivity. For instance, there is a higher level of HIV-1 protein transcriptions and elevated reverse transcriptase activity within Mtb granulomas than in other areas [68], and the enhancement of HIV replication in the blood of HIV-Mtb-coinfected individuals [69]. Additionally, Mtb can reactivate latent-HIV in T cells through the downstream effects of TLR2 [70]. However, there is a paucity of data investigating toll-like receptors’ antimicrobial and pathogenicity activity against the coinfection of these two pathogens. Previous studies provide compelling evidence of TLR2 in the pathogenesis of HIV-Mtb coinfection, but much less is known about the roles of other TLRs [58,71]. In this review, we wish to shed light on the host activity of endosomal TLRs 3, 7, and 9, in response to Mtb infection in healthy and HIV-coinfected individuals.

## 4. Toll-Like Receptors

Toll-like receptors (TLRs), among other receptors such as nucleotide-binding oligomerization (NOD)-like receptors (NLRs), RIG-I-like receptors, V-type lectin receptors, absent in melanoma 2(AIM2)-like receptors, and human oligoadenylate synthase (OAS)-like receptors, are Pattern Recognition Receptors (PRRs) that are expressed in innate immune cell [72]. These PRRs function in the recognition of a highly conserved molecular structure known as pathogen-associated molecular patterns (PAMPs) present on microbes [72]. TLRs were first discovered in the mid-1990s and have been studied extensively since [73,74]. TLRs are type I transmembrane proteins composed of three major domains: an extracellular domain consisting of leucine-rich repeats which facilitates the binding of PAMPs, a lipophilic transmembrane domain, and intracellular Toll-interleukin 1 receptor (TIR) domains required for interaction with downstream adaptor proteins [75]. The TLRs in vertebrate are classified into six families based on sequence homology. They are TLR1 (1,2,6,10), TRL3, TLR4, TLR5, TLR7 (7,8,9), and TLR11 (11,12,13) [75,76]. They are further classified based on their locations. TLRs expressed on the cell surface consist of TLR1, TLR2, TLR4, TLR5, TLR6, and TLR10, whereas intracellular TLRs, particularly expressed in endosomes, lysosomes, and endolysosomes, are composed of TLR3, TLR7, TLR8, TLR9, TLR11, TLR12, and TLR13 [76,77]. Interestingly, TLR3 is found in both intracellular locations and on the plasma membrane of human astrocytes [78].

The microbial molecular recognition pattern of TLRs is dictated by their residing locations. Cell surface TLRs primarily recognize the membrane components of microbes—namely, lipids, lipoproteins, and proteins [75]. On the other hand, intracellular TLRs mainly recognize microbial nucleic acids [75,76,77]. Upon activation, these receptors trigger the activation of cascades downstream, leading to the induction of immune response and cytokine production [79]. The signal transduction cascade of TLRs is initiated with the ligand recognition, resulting in the conformational change in the TIR domain, allowing the interaction of signal adaptor molecules—namely, myeloid differentiation primary response protein 88 (MyD88), TIR domain-containing adaptor inducing INF-(TRIF), and TRIF-related adaptor molecular (TRAM) [80]. The activation of adaptor molecules subsequently recruits and activates various kinases, especially Interleukin-1 receptor-associated kinases (IRAKs), TBK1, and inhibitor of NF-κB kinase ε; and ubiquitin ligases such as TNF receptor-associated factor (TRAF) 6 and Pellino1 [80,81]. Lastly, depending upon their individual pathway, these kinases or ubiquitin ligase will either release the major transcription factors NF-κB, specifically the RelA and p50 domain, from the inhibitor proteins known as IκB or translocate Interferon-regulatory factors (IRFs) into the nucleus for pro-inflammatory protein expression [82,83].

## 5. Toll-Like Receptor 3 and Mtb

**Figure 1:**
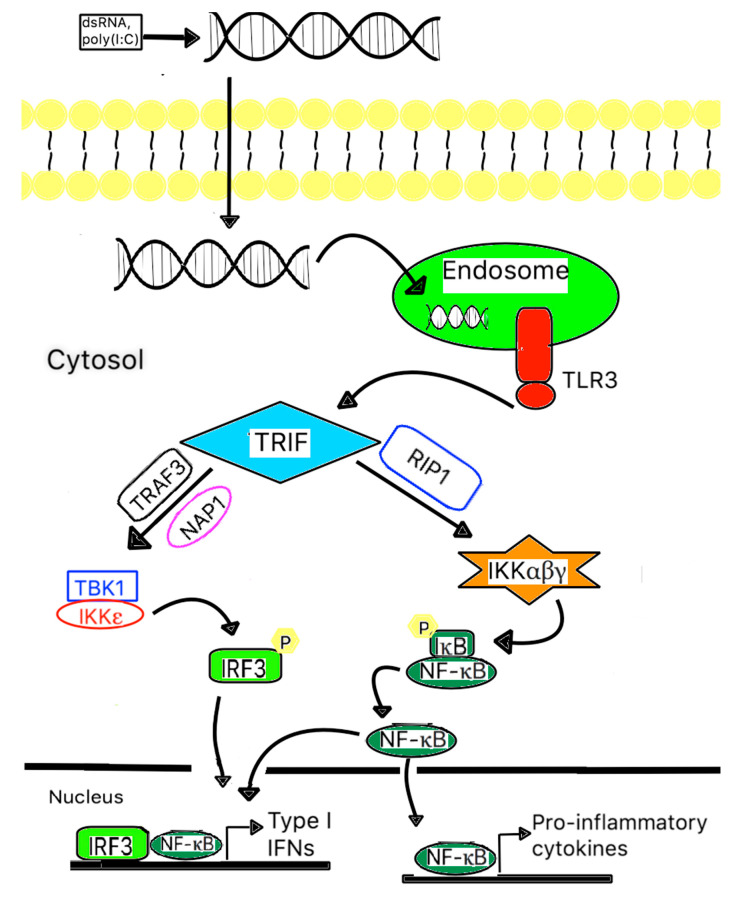
A scheme of TLR signaling pathways related to mycobacterial recognition. TLR3 is expressed on the endosomal membrane of cells. Uptake of either extracellular dsRNA or poly (I:C) into the cells and subsequent binding to TLR3 leads to the dimerization of TLR3, followed by recruitment of the adaptor protein TICAM-1/TRIF via a transient association with the TIR domain of TLR3. Subsequently, TICAM-1/TRIF will form a complex with RIP1, where it activates IKK and results in the activation of NF-B. TICAM-1/TRIF also forms a complex with TRAF3 and NAP1, where they recruit and activate TBK1 and IKK, resulting in the phosphorylation of IRF3. Both activated NF-kB and phosphorylated IRF3 are translocated into the nucleus and induce type I IFNs and other pro-inflammatory cytokine gene expression.

In general, TLR3 is expressed in various cell types, including myeloid dendritic cells, macrophages, Natural Killer cells, mast cells, neuronal cells, fibroblasts, endothelial cells, astrocytes, and epithelial cells [78,83]. It is responsible for recognizing intracellular viral or host RNA, particularly double-stranded RNA, derived from infected or damaged cells [68]. Unlike the rest of the TLRs, which utilize the MyD88-dependent pathway, TLR3 is facilitated through the TRIF-dependent pathway [84]. Upon activation, TLR3 recruits adaptor protein TRIF, which subsequently recruits TRAF3, TBK1, and IKKε and results in bifurcation effects [83,85]. The first one is the phosphorylation of interferon-regulatory factor 3 (IRF3), with the subsequent dimerization of phosphorylated-IRF3. The second one releases NF-κB from its inhibitor. The translocation of dimerized-IRF3 and activated NF-κB into the nucleus stimulates the immune response through the induction of type I IFN (α/β) expression, other cytokine and chemokine production, and dendritic cell maturation [75,85]. A schematic of signal transduction events is illustrated in Figure 1. Many studies have found that prolonged type I IFN signaling could be disastrous to the immune response by the induction of anti-inflammatory cytokines IL-10 and programmed death ligand (PDL) and hinder the responsiveness of a potent anti-mycobacterial molecule IFN-γ [86,87,88,89].

Nevertheless, the function of TLR3’s anti-mycobacterial properties remains un-elucidated and controversial. For instance, polyinosine-deoxycytidylic acid (poly I:C), a ligand of TLR3, stimulated autophagy by promoting the fusion of mycobacterial phagosomes with autophagosomes, thereby reducing the mycobacterial survival in mouse macrophages [90]. Additionally, Zaks et al. observed that vaccine adjuvants prepared with TLR3 agonists effectively cross-prime CD8+ T cells; thus, they promoted protective cell-mediated immunity against Mtb [91]. In contrast, Antonelli et al. and Huang et al. found that the stimulation of the TLR3 pathway using intranasal poly (I:C) provides a supportive environment for mycobacterial growth by enhancing triacylglyceride retention and reducing lipolysis in the macrophages of H37Rv-infected mice. On that note, the poly (I:C)-treated group was found to have more severe lung pathology than the control group [92,93]. Furthermore, Bai et al. demonstrated that the activation of TLR3 by mycobacterial RNA induces the production of IL-10 in BCG-mice macrophages via the PI3K/AKT signaling pathway. Correspondingly, TLR3 knockout mice exhibited a significant production of IL-12 and IFN-γ-secreting Th1 cells while restricting anti-inflammatory IL-10 production, thereby curtailing the bacterial burden and tissue damage [84,94]. Additionally, double-knockout TLR3 mice had elevated Th1 cell numbers in the spleen and reduced bacterial burden and tissue destruction in the lung when exposed to BCG [94]. Nevertheless, the aforementioned studies are limited to animal models, which may not accurately reflect humans’ responses. Not to mention that various cell types respond differently to TLR3 ligands due to the difference in the TLR3 expression level within the particular cell type. For instance, Jelinek et al. found that NK cells, B cells, and peritoneal macrophages expressed zero, low, and moderate amounts of TLR3, respectively [95]. Therefore, more future studies are necessary to elucidate the roles of TLR3 in human immune cells in response to Mtb infection during the acute and latent phase.

## 6. TLR3 vs. HIV and HIV-Mtb-Coinfection

The antiviral activity of TLR3 has remained unclear, and the outcome is a dependent variable of viral pathogens [96,97,98,99,100]. Similarly, the role of TLR3 in HIV and SIV pathogenesis has been investigated with conflicting results. TLR3 stimulates SIV promoters in macaques, but TLR3 ligands inhibit SIV replication [101]. Growing evidence supports that the activation of TLR3 can reactivate latent-HIV in reservoir cells by utilizing the IRF3-pathway in human microglial cells and the up-regulation of IL-6 in the human brain endothelium, resulting in endothelial inflammation and blood brain-barrier dysfunction in HIV patients [102,103,104]. Furthermore, the loss of TLR3 function by the TLR3/dsRNA complex inhibitor resulted in the suppression of HIV-induced endothelial IL-6 production mainly through the downregulation of the TAT1/JNK pathway [104]. Other evidence suggests that selective TLR3 activation promotes the production of type I IFNs, β-chemokines, and miRNA-155, which preferentially target 3′UTR of HIV-1 transcript, leading to a significant decline in HIV-1 infection in macrophages while enhancing the development of HIV-specific CD4+ and CD8+ cytotoxic T lymphocytes in humanized mice [103,105,106,107]. Therefore, to blunt the anti-HIV effects of TLR3, HIV-1 inhibits TLR3 signaling through blocking the phosphorylation of IRF3 and IRF7 and expresses significantly less HIV-restricted miRNAs [87]. In the same study, low levels of UNC93B1, a polytopic protein in the endoplasmic reticulum that interacts with the transmembrane domains of TLR3 with the subsequent loss of the TLR3-dependent production of type I IFNs, was observed in HIV-infected individuals [108]. Interestingly, the gene expression of TLR3 is not significantly different in chronic HIV-1 infected and healthy human macrophages, but it is markedly expressed in advanced stages [102,108,109]. Therefore, it is postulated that the persistent activation of TLR3 can contribute to the progression toward AIDS.

Unfortunately, the roles of TLR3 in the dual infection of HIV and Mtb are not clear due to the paucity of data. Nonetheless, a recent study had found a significant downregulation of TLR3 in HIV-Mtb coinfection compared to healthy and HIV-infected groups [110]. In the same study, the TLR3 gene expression is downregulated to a greater extent in Mtb-infected individuals with systemic and neurological symptoms compared to HIV-Mtb-coinfected individuals [110]. Thus, it is hypothesized that TLR3 has antimicrobial properties against HIV-Mtb coinfection, and the downregulation of TLR3 will diminish the immune responses, enabling HIV replication and facilitating the dissemination of TB [110].

## 7. Toll-Like Receptor 7 and 9 and Mtb

**Figure 2:**
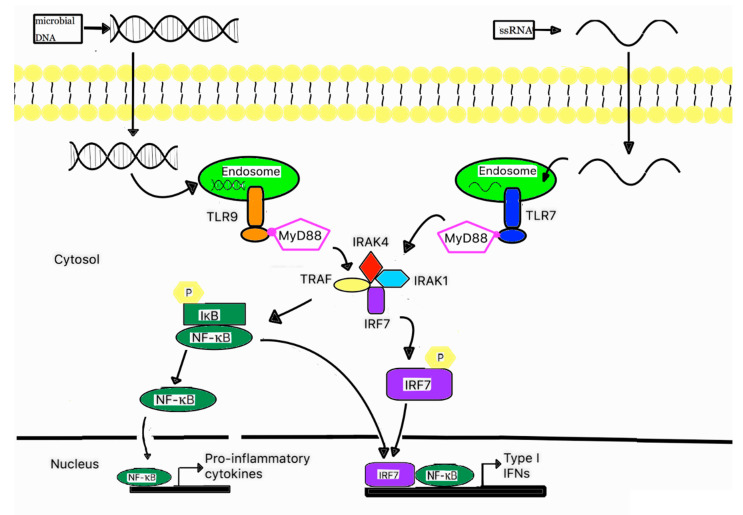
A schematic signaling pathway of TLR7 and 9. Plasmacytoid dendritic cells abundantly express TLR7 and TLR9 on their endosomal membranes, TLR7 and TLR9 are responsible for recognizing ssRNA and unmethylated CpG region of DNA. Once activated, TLR7 and TLR9 recruit adaptor protein MyD88, which subsequently forms a complex with IRAK4, TRAF6, IRAK1, and IRAF7. This complex can activate NF-kB and phosphorylate IRF7. Then, the activated NF-kB and phosphorylated IRF7 are translocated into the nucleus, where they induce the gene expression of pro-inflammatory cytokines and type I IFNs.

TLR7 and TLR9 are known to be expressed in macrophages, and are even more abundant in plasmacytoid dendritic cells and respond to the presence of foreign single-stranded RNA (ssRNA) and the unmethylated CpG region of DNA, respectively [111,112,113,114]. Interestingly, unmethylated CpG is usually found in bacterial DNA due to the absence of CpG methylation enzymes, whereas the CpG in mammalian DNA is methylated [115]. Therefore, it is reasonable that TLR9 does not recognize self-DNA. In fact, TLR9 is one of the PRRs that are most frequently associated with the recognition of Mtb PAMPs [116]. Both TLR7 and TLR9 follow the MyD88-dependent pathway; hence, they recruit adaptor protein MyD88 upon activation. Subsequently, MyD88 recruits and forms a complex with other downstream molecules—namely IRAK4, IRAK1, TRAF6, and IRF7. Similar to the TRIF-dependent pathway, this MyD88 complex performs a dual function. One is the translocation of activated NF-κB, while another function is the translocation of phosphorylated IRF7 to the nucleus, as shown in Figure 2 [77]. The MyD88-dependent pathway promotes the expression of pro-inflammatory cytokines, including TNF-α and IL-6, and type I IFNs [77,117,118].

The roles of TLR7 in providing host cells immunity against Mtb appear to be consistent throughout the literature, despite the limited number of studies. For instance, TLR7 activation can trigger autophagy [119], through the regulation of autophagy positive and negative effectors extracellular-signal receptor kinase (ERK) and p38, respectively, in RAW264.7 macrophages, rendering resistance against Mtb [120]. Furthermore, Bakhru et al. revealed that both TLR7 and 9 are capable of inducing MHCII expression through the activation of mitogen-activated protein kinase (MAPK) and AP-1/CREB. They can also simultaneously inhibit the ubiquitination of MHCII by down-regulating MARCH1, a ubiquitin ligase, in macrophages. In the same study, TLR7 and 9 are responsible for reducing the anti-inflammatory cytokine IL-10, which is secreted by Treg and functioning in the downregulation of MHCII macrophages [121]. Together, TLR7 and 9 allow more MHCII to be available to optimally interact with and activate CD4+ T cells against the Mtb invasion [122]. Evidence also suggests that TLR9 indirectly facilitates cell-mediated immunity, which promotes the priming and maturation of CD8+ T cells against Mtb antigens, by TLR9-induced type I IFNs [102]. Conversely, another study has demonstrated the activation of TLR9 in the pathogenesis of Mtb in vitro by a significant increase in the Treg and IL-10 expression, which is responsible for dampening the immune response and facilitating the reactivation and dissemination of Mtb. On that note, the blockage of TLR9 resulted in a reduction in Treg and IL-10, with increased IFN-γ production, cumulatively providing a protective effect against Mtb [123]. Intriguingly, TLR9 activation leads to a dichotomous dose-dependent effect, with low-level activation leading to a primarily Th1 response, while increased activation suppresses the immune response [103]. The co-activation of TLR9 with another TLR, specifically TLR2, is also investigated with contradicting results. A study demonstrated that the co-activation of TLR2 and TLR9 produces favorable effects conferring immunity against Mtb, while other studies found the opposite results [124,125]. However, the double knockout TLR2/TLR9 does not consistently enhance Mtb susceptibility [12,125]. This finding suggests that host cell immunity against Mtb involves highly complex pathways with differential responses from other PRRs within the innate immune system.

## 8. TLR7 and 9 vs. HIV and Mtb-HIV Coinfection

As mentioned above, the primary role of TLR7 and 9 is conferring immunity against viral pathogens. Surprisingly, the role of TLR7 in response to HIV is the dependence of time. In the early stage of HIV infection, the activation of TLR7 results in ramping up the production of type I IFNs, and the downregulation of HIV coreceptors CCR5 and CXCR4 in CD4+ T cells [126]. Thus, the stimulation of TLR7 suppresses HIV viral production and amplifies the antiviral response from macrophages, activated T cells, and monocytes by inducing type I IFN production, while blocking TLR7 abolishes these anti-HIV effects [126,127]. However, in the later stage of infection, TLR7 plays a pivotal role in the pathogenesis and disease advancement of HIV. Due to the nature of chronic infection and persistent activation, the TLR7-dependent pathway continuously induces TNF-related apoptosis-inducing ligand (TRAIL) in monocytes [128] and CD4+ T cells, which inadvertently induces apoptosis in uninfected CD4+ T cells, resulting in a declining number of CD4+ T cells [129]. In the same manner, the continuous activation of TLR7 in HIV-infected cells results in a marked reduction in IL-2 and IFN-γ proteins and a sustained increase in the intracellular calcium, ultimately leading to CD4+ T cell anergy [130]. Therefore, TLR7 seems to provide a dualistic function of preventing catastrophic effects of overwhelming infection in host cells while continuously preserving HIV replication in reservoir cells [127]. These findings can lead to speculation on how continuously activated-TLR7 blunts anti-HIV and anti-Mtb effects via the dysregulation of CD4+ T cells and IFN-γ in HIV-Mtb-coinfected individuals.

It is not well understood how TLR9 responds in HIV-infected individuals. One study concluded that the activation of TLR9 induces the activation of NF-κB, but inadvertently increases the initiation and elongation of viral transcription, thereby reactivating latent HIV in human CD4+ T cells in vitro [131]. On the contrary, another study revealed that the stimulation of TLR9 in pDC enhances the production of type I IFNs, thus conferring immunity against HIV [132]. To circumvent this immune response, HIV abrogates the production of type I IFNs indirectly by increasing the IL-10 level and directly by disrupting the function of pDC [132,133,134]. Furthermore, HIV gp120, an envelope glycoprotein, inhibits TLR9-mediated pDC maturation, activation, and cytokine production by reducing the expression of IRF7 and type I IFNs [135,136]. However, many studies have provided conflicting results on how HIV gp120 mediates the IFN-α production in plasmacytoid dendritic cells [137,138,139]. Since TLR9 is frequently associated with the recognition of Mtb, it has been found that the persistent activation of TLR9 due to the chronic infection of Mtb results in the steady production of many cytokines—namely, IL-1a, IL-1B, IL-6, IL8, TNFs, and IFNs. Those cytokines bind to a highly conversed HIV long terminal repeat (LTR), which, in turn, activates HIV transcription [140]. Besides, the co-stimulation of TLR7 and 9 in dendritic cells can result in a transient increase in IFN-γ-induced protein 10 (IP-10), which is a potent recruiter of Th1 cells, promoting temporary cellular immunity against HIV [141]. However, the co-stimulation inadvertently elevates IL-10 and IFN-α, which is also a stimulator of IL-10, leading to an adversarial influence in host cell immunity against HIV and Mtb through the suppression of T-cell function and IFN-γ [88,142,143,144]. Therefore, it is hypothesized that the persistent activation of both TLR 7 and 9 results in the depletion of monocytes, macrophages, CD4+ T cells, and IFN-γ, rendering host cells susceptible to infection and the disease progression of HIV and Mtb, especially in HIV-Mtb coinfection.

Moreover, the adaptor proteins also play a pivotal role in TLR-mediated immune response. Defective adaptor proteins MyD88 and IRAK4 inhibit IL-1β, MCP-1, and IP-10 cytokine production and induce TNF-α and IL-10 cytokine production by monocytes in HIV-Mtb-coinfected individuals, thereby impeding both anti-mycobacterial and anti-HIV response [145]. Likewise, it was shown that silencing MyD88, IRAK1, and TRAF6 is capable of downregulating NF-κB and nuclear factor of activated T cells 5 (NFAT5), which binds to HIV-1 (LTR) and facilitates HIV viral transcription, after Mtb infection [110]. However, the complex mechanisms of TLR7 and 9 involving their respective adaptor proteins in HIV-Mtb-coinfected individuals remain to be understood entirely.

## 9. Conclusions

HIV and Mtb remain two of the most infectious agents contributing to mortality worldwide. Previous studies support the synergistic effects that HIV and Mtb exert on the immune system to circumvent host cell responses and accelerate disease progression. Pattern recognition receptors, particularly toll-like receptors, of the innate immune system play a pivotal role in mounting immune response, but are also implicated in the pathogenesis of HIV and Mtb coinfection. Among TLRs, TLR3, 7, and 9 are expressed intracellularly and respond to foreign nucleic acids. Their immune response is facilitated primarily by the production of type I IFN (α/β) expression, other cytokines, and chemokines. However, these responses appear to confer cell immunity in acute infection but accelerate disease in the chronic infection of HIV and Mtb infection. Unfortunately, much is unknown about the disease pathogenesis mechanism that each intracellular TLR undertakes in light of HIV-Mtb coinfection. In addition, the pathogenesis and host immune response in chronic HIV-Mtb coinfection involves the perplexing interconnectedness of other pathways, constitutively activating TLR3, 7, and 9. With limited available data, it is imperative for future studies to investigate the functional roles of endosomal TLRs in HIV-Mtb-coinfection with strategies to develop the short-term upregulation of these PRRs to control the infection during the acute phase effectively and put a halt to their continuous activation in the later stage to delay disease progression. Finally, with the better understanding of endoplasmic TLRs, effective interventions and vaccines can be developed against these two deadly diseases.
